# Preconceptional Folate Supplementation and the Risk of Spontaneous Preterm Birth: A Cohort Study

**DOI:** 10.1371/journal.pmed.1000061

**Published:** 2009-05-12

**Authors:** Radek Bukowski, Fergal D. Malone, Flint T. Porter, David A. Nyberg, Christine H. Comstock, Gary D. V. Hankins, Keith Eddleman, Susan J. Gross, Lorraine Dugoff, Sabrina D. Craigo, Ilan E. Timor-Tritsch, Stephen R. Carr, Honor M. Wolfe, Mary E. D'Alton

**Affiliations:** 1 University of Texas Medical Branch, Department of Obstetrics and Gynecology, Galveston, Texas, United States of America; 2 Royal College of Surgeons in Ireland, Department of Obstetrics and Gynecology, Dublin, Ireland; 3 University of Utah, Salt Lake City, Utah, United States of America; 4 The Fetal & Women's Center of Arizona, Scottsdale, Arizona, United States of America; 5 William Beaumont Hospital, Fetal Imaging Department, Royal Oak, Michigan, United States of America; 6 Mount Sinai Medical Center, Department of Obstetrics and Gynecology, New York, New York, United States of America; 7 Montefiore Medical Center–Albert Einstein College of Medicine, Department of Obstetrics & Gynecology and Women's Health, Bronx, New York, United States of America; 8 University of Colorado Health Sciences Center, Department of Obstetrics and Gynecology, Denver, Colorado, United States of America; 9 Tufts-New England Medical Center, Department of Obstetrics and Gynecology, Boston, Massachusetts, United States of America; 10 New York University Medical Center, School of Medicine, Department of Obstetrics and Gynecology, New York, New York, United States of America; 11 Brown University/Women & Infants' Hospital, Department of Obstetrics and Gynecology, Providence, Rhode Island, United States of America; 12 University of North Carolina at Chapel Hill, Department of Obstetrics and Gynecology, Chapel Hill, North Carolina, United States of America; 13 Columbia University Medical Center, Department of Obstetrics and Gynecology, Maternal-Fetal Medicine, New York, New York, United States of America; University of Queensland Centre for Clinical Research, Australia

## Abstract

**Background:**

Low plasma folate concentrations in pregnancy are associated with preterm birth. Here we show an association between preconceptional folate supplementation and the risk of spontaneous preterm birth.

**Methods and Findings:**

In a cohort of 34,480 low-risk singleton pregnancies enrolled in a study of aneuploidy risk, preconceptional folate supplementation was prospectively recorded in the first trimester of pregnancy. Duration of pregnancy was estimated based on first trimester ultrasound examination. Natural length of pregnancy was defined as gestational age at delivery in pregnancies with no medical or obstetrical complications that may have constituted an indication for delivery. Spontaneous preterm birth was defined as duration of pregnancy between 20 and 37 wk without those complications. The association between preconceptional folate supplementation and the risk of spontaneous preterm birth was evaluated using survival analysis. Comparing to no supplementation, preconceptional folate supplementation for 1 y or longer was associated with a 70% decrease in the risk of spontaneous preterm delivery between 20 and 28 wk (41 [0.27%] versus 4 [0.04%] spontaneous preterm births, respectively; HR 0.22, 95% confidence interval [CI] 0.08–0.61, *p* = 0.004) and a 50% decrease in the risk of spontaneous preterm delivery between 28 and 32 wk (58 [0.38%] versus 12 [0.18%] preterm birth, respectively; HR 0.45, 95% CI 0.24–0.83, *p* = 0.010). Adjustment for maternal characteristics age, race, body mass index, education, marital status, smoking, parity, and history of prior preterm birth did not have a material effect on the association between folate supplementation for 1 y or longer and spontaneous preterm birth between 20 and 28, and 28 to 32 wk (adjusted HR 0.31, 95% CI 0.11–0.90, *p* = 0.031 and 0.53, 0.28–0.99, *p* = 0.046, respectively). Preconceptional folate supplementation was not significantly associated with the risk of spontaneous preterm birth beyond 32 wk. The association between shorter duration (<1 y) of preconceptional folate supplementation and the risk of spontaneous preterm birth was not significant after adjustment for maternal characteristics. However, the risk of spontaneous preterm birth decreased with the duration of preconceptional folate supplementation (test for trend of survivor functions, *p* = 0.01) and was the lowest in women who used folate supplementation for 1 y or longer. There was also no significant association with other complications of pregnancy studied after adjustment for maternal characteristics.

**Conclusions:**

Preconceptional folate supplementation is associated with a 50%–70% reduction in the incidence of early spontaneous preterm birth. The risk of early spontaneous preterm birth is inversely proportional to the duration of preconceptional folate supplementation. Preconceptional folate supplementation was specifically related to early spontaneous preterm birth and not associated with other complications of pregnancy.

## Abstract 2

In an analysis of a cohort of pregnant women, Radek Bukowski and colleagues describe an association between taking folic acid supplements and a reduction in the risk of preterm birth.

## Editors' Summary

**Background:**

Most pregnancies last about 40 weeks, but sometimes the new family member arrives early. Every year, half a million babies in the United States (12.5% of all babies) are born prematurely (before 37 completed weeks of pregnancy). Sadly, premature babies are more likely to die than full-term babies and many have short- and/or long-term health problems. Premature babies often have breathing problems, they are susceptible to life-threatening infections, and they are more likely to have learning and developmental disabilities than those born on time. The severity of these health problems depends on the degree of prematurity—preterm babies born between 34 and 36 weeks of pregnancy rarely develop severe disabilities, but a quarter of babies born before 28 weeks of pregnancy develop serious lasting disabilities and half have learning and behavioral problems. Although doctors have identified some risk factors for early delivery (for example, smoking), it is impossible to predict who will have an early birth and there is no effective way to prevent preterm births.

**Why Was This Study Done?:**

Some researchers think that folate supplements may prevent preterm births. Folate (folic acid), a vitamin found in leafy green vegetables, fruits, and dried beans, helps to prevent neural tube birth defects. Consequently, women are encouraged to take folic acid supplements throughout (and preferably before) pregnancy and many governments now mandate that bread, pasta, and other grain products be fortified with folic acid to help women get sufficient folate. There is some evidence that women who deliver early have less folate in their blood than women who deliver at term. Furthermore, folate supplementation during pregnancy has increased the length of pregnancy in some but not all clinical trials. A possible explanation for these mixed results is that the duration of pregnancy reflects conditions in the earliest stages of pregnancy or before conception and that folate supplementation needs to start before conception to reduce the risk of preterm birth. In this study, the researchers test this idea by analyzing data collected from nearly 35,000 pregnant women enrolled in a study that was originally designed to investigate screening for Down's syndrome.

**What Did the Researchers Do and Find?:**

During the first three months of their pregnancy, the women were asked whether they had taken folate supplements before conception. The duration of each pregnancy was estimated from ultrasound measurements taken early in the pregnancy and from the time of delivery. During the study, 1,658 women had spontaneous preterm deliveries before 37 weeks and 160 delivered before 32 weeks. After allowing for other maternal characteristics that might have affected the likelihood of preterm delivery, the risk of spontaneous preterm delivery between 20 and 28 weeks was 70% lower in women who took folate supplements for more than a year before becoming pregnant than in women who didn't take a supplement. Long-term folate supplementation also reduced the risk of preterm delivery between 28 and 32 weeks by 50% but did not affect the risk of preterm birth beyond 32 weeks. Folate supplementation for less than a year before conception did not reduce the risk of preterm birth, and folate supplementation was not associated with any other complications of pregnancy.

**What Do These Findings Mean?:**

These findings show that folate supplementation for a year or more before conception is associated with a 50%–70% decrease in early (but not late) spontaneous preterm births and that the longer a woman takes folate supplements before becoming pregnant, the lower her risk of a preterm birth. Although the researchers allowed for maternal characteristics that might have affected the duration of pregnancy, it is possible that folate supplementation may not be responsible for the reduction in preterm birth risk seen in this study. For example, taking folate supplements may be a marker of healthy behavior and the women taking the supplements might have been doing something else that was reducing their risk of preterm birth. However, despite this and other limitations of this study, these findings suggest that long-term folate supplementation before conception is worth investigating further as a potential way to prevent preterm births.

**Additional Information:**

Please access these Web sites via the online version of this summary at http://dx.doi.org/10.1371/journal.pmed.1000061.

- This study is further discussed in a *PLoS Medicine* Perspective by Nicholas Fisk
- The MedlinePlus encyclopedia contains a page on premature babies (in English and Spanish); MedlinePlus provides links to other information on premature babies (in English and Spanish)
- The US National Institute of Child Health and Human Development provides information on preterm labor and birth
- The March of Dimes, a nonprofit organization for pregnancy and baby health, provides information on preterm birth and on folic acid (in English and Spanish)
- The Nemours Foundation, another nonprofit organization for child health, also provides information on premature babies (in English and Spanish)
- The US Office of Dietary Supplements has a fact sheet on folate

## Introduction

Preterm birth is the main cause of neonatal mortality and short- and long-term morbidity. Neonatal mortality and morbidity are inversely correlated with gestational age at delivery, with the majority of those adverse outcomes associated with early preterm birth (i.e., delivery before 32 wk gestation) [1]. As a consequence, societal costs of preterm births in the US exceed US$26 billion a year [1].

Unfortunately there are no effective methods of prevention or treatment of preterm birth. The most promising intervention—progesterone supplementation—to date has been shown to be effective only in a small subset of women at risk for preterm birth [2],[3]: in women with prior preterm birth or with a short cervix (≤15 mm), constituting respectively 3.3% and 1.7% of pregnancies at risk [4],[5].

Several studies demonstrated an association between lower concentrations of folate and shorter duration of pregnancy. Observational studies have showed that women delivering preterm have lower concentrations of folate in plasma or red blood cells [6]–[9]. Results of interventional trials of folate supplementation during pregnancy are less conclusive. Supplementation of folate during pregnancy resulted in longer duration of pregnancy in some [10],[11] but not in all studies [12]–[14].

However, a growing body of evidence of human studies and animal models suggests that duration of pregnancy may be the ultimate consequence of conditions in the very earliest stages of pregnancy or before conception [15]–[17]. We tested the hypothesis that preconceptional folate supplementation is associated with reduction in the risk of spontaneous preterm birth.

## Methods

### Study Population

In a secondary analysis of a previously described prospective cohort study [18], we studied 34,480 women who delivered singleton pregnancies between 20 wk 0 d and 42 wk 0 d. The original Down syndrome screening study was conducted at 15 US centers from October 1999 to December 2002 (Table S2). The inclusion criteria were a maternal age of 16 y or older, singleton live fetus, and a fetal crown–rump length corresponding to a gestational age of 10 wk 3 d through 13 wk 6 d at enrollment. Women were excluded from the study if they had undergone prior measurement of nuchal translucency or if anencephaly was diagnosed in the fetus.

Extensive information on patients' demographics, medical and obstetrical history, socioeconomic status measures, and exposures during pregnancy were recorded. Copies of medical records were reviewed in all cases where parents reported a possible neonatal medical problem, in all Down syndrome screen–positive cases without karyotype results, and in a 10% random sample of all other enrolled cases by a single abstractor. Institutional review board approval was obtained in all participating centers, and all participants gave written informed consent.

Out of 42,367 patients approached, 38,033 patients were enrolled. Of those enrolled 189 (0.5%) had missing data on folate supplementation, 430 (1.1%) missing confounder data, 529 (1.4%) gestational age at delivery >42 wk, 2,103 (5.5%) missing gestational age at delivery, and 302 (0.8%) miscarried or aborted pregnancy between enrollment and 20 wk. Complete data on maternal characteristics, exposures, pregnancy complications, and outcomes were available for 34,480 (91.4%) of the remaining pregnancies.

### Definition of Maternal Characteristics

Duration of consistent preconceptional folate supplementation, with or without multivitamins, was self-reported in the first trimester of pregnancy at the time of enrollment and patients were followed until delivery. Duration of preconceptional folate supplementation was reported as: <1, 1–2, 3–4, 5–6, 7–11, and >12 mo and was categorized into three groups: ≥1 y, <1 y, and no preconceptional supplementation.

Maternal age was defined as maternal age at the time of birth and categorized into <35 and ≥35 y to reflect advanced maternal age associated with increased risk of adverse pregnancy outcomes. Race and ethnicity were self-reported by mother in the first trimester of pregnancy. Maternal height and weight obtained in the first trimester of pregnancy, respectively in cm and kg, were included in calculation of body mass index. Body mass index was categorized into three groups: <20, 20–30, and >30. Maternal education level was self-reported in total completed years of education and categorized into ≤12 or >12 y. Marital status was defined as married or not married. Both education and marital status were used as measures of socioeconomic status. Smoking was defined as a self-reported smoking status of the woman in the first trimester. Prior preterm birth was defined as prior pregnancy lasting less than 37 wk 0 d.

### Definition of Preterm Birth

Duration of pregnancy in all women was based on ultrasound measurement of crown–rump length in the first trimester. Preterm birth was defined as duration of pregnancy between 20 wk 0 d and 36 wk and 6 d [1].

Spontaneous preterm birth was defined as preterm birth not associated with medical or obstetrical complications constituting indications for preterm delivery. Those indications included: chromosomal abnormalities, structural malformations, stillbirth, termination of pregnancy, pregnancy induced hypertension, preeclampsia, chronic hypertension, gestational or pregestational diabetes, placenta previa, and placental abruption.

### Secondary Outcomes

Small for gestational age was an infant with birth weight below the 10th percentile for gestational age [19]. Preeclampsia and placental abruption were self-reported.

### Statistical Analyses

Duration of preconceptional folate supplementation, categorized ≥1 y, <1 y, and no supplementation, was compared across maternal and obstetric characteristics. Univariate comparison of continuous variables was performed using the Kruskal-Wallis test and of categorical data using the Chi-square or Fisher exact tests.

All *p*-values are two-sided. Statistical significance is assumed at *p*<0.05.

The risk of spontaneous preterm birth between the categories of duration of preconceptional folate supplementation was compared using time-to-event analysis. The gestational age from 20 wk onward was used as the time scale. Gestational age at delivery between 20 wk 0 d and 36 wk 6 d was taken as time of the event in the absence of indications for delivery or as the time of censoring in either their presence or if gestational age at delivery was 37 wk 0 d or later. This approach allows assessment of the variation in relative risk of spontaneous preterm birth with gestational age.

The time to event data are presented as a cumulative percentage of the event, as recommended for rare events [20]. The univariate comparisons were made using a log-rank test. The crude and adjusted for maternal characteristics and risk factors for preterm birth hazard ratios (HRs) were obtained using Cox proportional hazard regression [21]. The risk of spontaneous preterm birth was adjusted for maternal age, race and ethnicity, body mass index in the first trimester of pregnancy, education, marital status, smoking status, parity, and history of prior preterm birth and recruitment center. The interaction terms between duration of preconceptional folate supplementation and categorized maternal characteristics and risks factors for preterm birth were assessed using Cox proportional hazard regression and multivariate logistic regression analyses. The proportional hazard assumption was tested using global and specific tests to identify categories of spontaneous preterm birth with constant HR [22]. The constancy of the log HR function was inspected visually, and identified intervals of preterm birth were tested using the proportional hazard assumption test of nonzero slope of the scaled Schoenfeld residuals of time. Goodness of fit was assessed using the test of Groennesby and Borgan [23]. Analysis of effect modification was performed using interaction terms in the proportional hazard regression and multivariable logistic regression for strata of each maternal characteristic adjusted for all other characteristics. The association between duration of preconceptional folate supplementation and the risk of preeclampsia, placental abruption, and delivery of small for gestational age infant were analyzed using multivariable logistic regression. Statistical analyses were performed using Stata 10 (Stata, http://www.stata.com/).

## Results

In the study population of 34,480 women, preconceptional folate supplementation was used by 6,777 (19.6%) for ≥1 y, by 12,444 (36.1%) for <1 y, and not at all by 15,259 (44.3%) (Table 1). Preconceptional folate supplementation was positively associated with maternal age, white race, being married, education duration above 12 y, and nulliparity, whereas it was negatively associated with body mass index, smoking, Hispanic and black ethnicity and race, and prior birth at term or preterm (Table 1). Preconceptional folate supplementation was associated with higher birth weight and lower rates of spontaneous preterm birth before 32 and 37 wk and preterm premature rupture of the membranes (PPROM) before 32 wk (Table 1).

**Table 1:** Maternal characteristics and pregnancy outcomes in relation to preconceptional folate supplementation in 34,480 singleton pregnancies.

Characteristics	Preconceptional Folate Supplementation			*p*-Value^a^
	None	<1 y	≥1 y	
***n*** ** (% of total)**	15,259 (44.3%)	12,444 (36.1%)	6,777 (19.6%)	N/A
**Age, y (median IQR)**	27.8 (23.5–32.5)	31.1 (27.1–34.5)	33.1 (29.4–36.3)	<0.0001
**BMI (median IQR)**	24.5 (21.7–28.4)	23.3 (21.2–26.4)	23.2 (21.2–26.3)	<0.0001
**Race/ethnicity, ** ***n*** ** (%)**				
White	7,672 (50.3)	9,931 (79.8)	5,906 (87.1)	<0.0001
Hispanic	5,674 (37.2)	1,501 (12.1)	386 (5.7)	<0.0001
Black	1,151 (7.5)	384 (3.1)	180 (2.7)	<0.0001
Asian	599 (3.9)	520 (4.2)	248 (3.7)	0.6
Native	95 (0.6)	73 (0.5)	37 (0.5)	0.5
Other	68 (0.5)	35 (0.3)	20 (0.3)	0.04
**Marital status, ** ***n*** ** (%)**				<0.0001
Not married	5,667 (37.1)	1,122 (9.0)	463 (6.8)	
Married	9,592 (62.9)	11,322 (91.0)	6,314 (93.2)	
**Education, ** ***n*** ** (%)**				<0.0001
≤12 y	6,769 (44.3)	1,663 (13.4)	608 (9.0)	
>12 y	8,496 (55.7)	10,781 (86.6)	6,169 (91.0)	
**Smoking status, ** ***n*** ** (%)**	1,181 (7.8)	289 (2.3)	149 (2.2)	<0.0001
**Obstetrical history, ** ***n*** ** (%)**				
Nulliparous	6,072 (39.8)	6,440 (51.8)	3,036 (44.8)	<0.0001
Parous with no prior preterm birth	8,040 (52.7)	5,290 (42.5)	3,276 (48.3)	<0.0001
Parous with one or more prior preterm births	1,147 (7.5)	714 (5.7)	465 (6.9)	0.002
**Duration of pregnancy, d (IQR)**	39.4 (38.6–40.3)	39.6 (38.6–40.3)	39.4 (38.6–40.3)	0.1
**Birth weight, g (IQR)**	3,345 (3033–3657)	3,375 (3062–3686)	3,375 (3061–3714)	0.0001
**Preterm birth, ** ***n*** ** (%)**				
<37 wk	1,263 (8.3)	894 (7.2)	503 (7.4)	0.005
<32 wk	253 (1.7)	147 (1.2)	73 (1.1)	<0.0001
Spontaneous <37 wk	790 (5.2)	558 (4.5)	310 (4.6)	0.017
Spontaneous <32 wk	99 (0.7)	46 (0.4)	16 (0.2)	<0.0001
Nonspontaneous^b^ <37 wk	473 (3.1)	336 (2.7)	193 (2.9)	0.2
Nonspontaneous^b^ <32 wk	154 (1.0)	101 0.8)	58 (0.9)	0.2
PPROM <37 wk	204 (1.3)	144 (1.2)	76 (1.1)	0.1
PPROM <32 wk	61 (0.4)	34 (0.3)	11 (0.2)	0.002

a Kruskal-Wallis test or test for trend as appropriate.

b Nonspontaneous indicates preterm birth associated with complications of pregnancy constituting indications for delivery.

**Figure 1:**
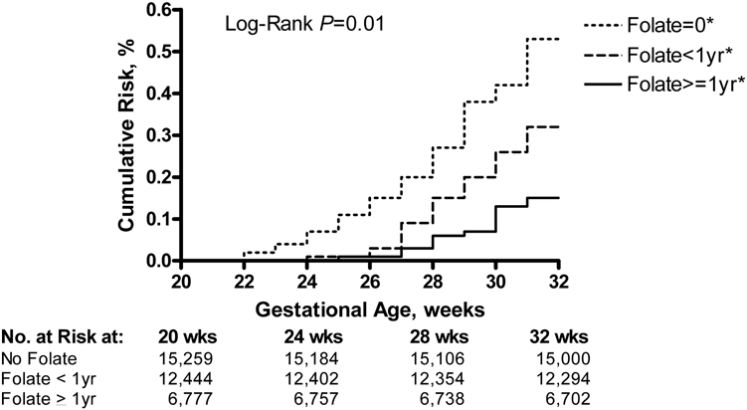
Cumulative risk of spontaneous preterm birth in relation to duration of preconceptional folate supplementation. Folate = 0*, no preconceptional folate supplementation; Folate <1 yr*, preconceptional folate supplementation for less than a year; Folate > = 1 yr*, preconceptional folate supplementation for a year or longer. *Test for trend of survivor functions, *p* = 0.01.

A total of 2,660 women (7.7%) delivered prematurely before 37 wk, and 473 (1.4%) before 32 wk. Spontaneous preterm birth occurred in 1,658 women (4.8%) before 37 wk and in 160 (0.5%) before 32 wk (Table 1). The risk of spontaneous preterm birth decreased with the duration of preconceptional folate supplementation (test for trend of survivor functions, *p* = 0.01; Figure 1) and was the lowest in women who used folate supplementation for a year or longer.

The protective effect of folate supplementation decreased with advancing gestational age (Figure 1). Compared to women without preconceptional folate supplementation, women with a year or longer of supplementation had 70% lower risk (0.27% and 0.04%, respectively), and women with supplementation shorter than a year had 50% (0.27% and 0.16%, respectively) lower risk of spontaneous preterm birth between 20 and 28 wk (Table 2) in unadjusted analysis. The crude risk of spontaneous preterm birth between 28 and 32 wk was 50% and 30% lower, respectively, for the two supplementation groups (0.38% and 0.18%; 0.38% and 0.23%, respectively). The risk of spontaneous preterm births between 32 and 37 wk was not significantly different between the folate supplementation groups (Table 2).

**Table 2:** Risk of spontaneous preterm birth and preconceptional folate supplementation.

Preterm Birth Gestational Age	No Folate		Folate <1 y		Folate ≥1 y		Folate <1 y				Folate ≥1 y			
	No. (%) (*n* = 15,259)	Incidence	No. (%) (*n* = 12,444)	Incidence	No. (%) (*n* = 6,777)	Incidence	Unadjusted HR (95% CI)	*p*-Value	Adjusted HR (95% CI)	*p*-Value	Unadjusted HR (95% CI)	*p*-Value	Adjusted HR (95% CI)	*p*-Value
**20–28 wk**	41 (0.27)	0.34	17 (0.14)	0.17	4 (0.04)	0.06	0.54 (0.31–0.93)	0.028	0.72 (0.40–1.28)	0.3	0.22 (0.08–0.61)	0.004	0.31 (0.11–0.90)	0.031
**28–32 wk**	58 (0.38)	0.96	29 (0.23)	0.59	12 (0.18)	0.45	0.67 (0.44–1.02)	0.061	0.73 (0.47–1.13)	0.2	0.45 (0.24–0.83)	0.010	0.53 (0.28–0.99)	0.046
**32–37 wk**	691 (4.53)	9.21	512 (4.16)	8.33	295 (4.35)	8.80	0.89 (0.80–1.00)	0.055	0.93 (0.83–1.06)	0.3	0.95 (0.83–1.09)	0.5	0.99 (0.85–1.15)	0.9

Incidence expressed per 1,000 women per week in a gestational age period (20–28, 28–32, or 32–37 wk). Risk estimates are calculated in comparison to the group without preconceptional folate supplementation. HR adjusted for maternal age, body mass index, race and ethnicity, educational level, marital status, smoking, parity and history of prior preterm birth and recruitment center. Number needed to treat (NNT) for folate supplementation ≥1 y is 435 women to prevent one case of spontaneous preterm birth between 20 and 28 wk, and 500 women to prevent one spontaneous preterm birth between 28 and 32 wk.

The proportional hazard assumption was violated for the gestational age interval from 20 to 37 wk, indicating that the risk of spontaneous preterm birth in relation to duration of folate supplementation varies during this interval (proportional hazard test *p*<0.0001 and *p* = 0.006 for duration of folate supplementation for more or less than a year, respectively). However, for gestational age intervals 20 to 28 (20 wk 0 d to 27 wk 6 d), 28 to 32 (28 wk 0 d to 31 wk 6 d) and 32 to 37 wk (32 wk 0 d to 36 wk 6 d) risks of spontaneous preterm birth were constant and proportional hazard assumption was met (proportional hazard tests *p*>0.2 for all). Hence, the preterm birth gestational age intervals evaluated in this study were identified by testing proportional hazard assumption, rather than selected arbitrarily and are the gestational age intervals with constant hazard ratios for spontaneous preterm birth.

Duration of preconceptional folate supplementation was also associated with significantly reduced risk of all preterm birth, spontaneous or not, in a similar manner (test for trend of survivor functions *p* = 0.004). Thus there was a significant association between duration of preconceptional folate supplementation and the risk of preterm birth before 32 wk when pregnancies with complications constituting indication for delivery were censored or not. However, there was no significant association between duration of preconceptional folate supplementation and the risk of non-spontaneous, complicated by obstetrical or medical conditions, preterm birth.

**Figure 2:**
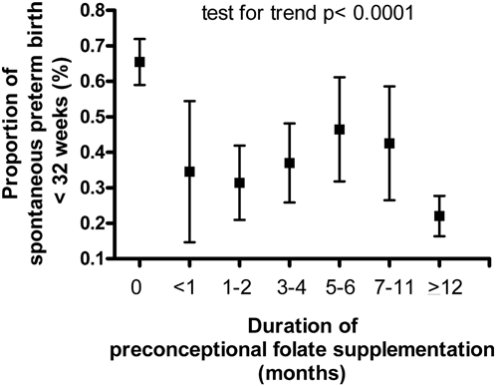
Risk of spontaneous preterm birth before 32 wk in relation to duration of preconceptional folate supplementation. Proportion (mean ± standard error) of spontaneous preterm births before 32 wk in women with no preconceptional folate supplementation and supplementation for <1, 1–2, 3–4, 5–6, 7–11, and >12 mo.

The association between duration of preconceptional folate supplementation and the risk of spontaneous preterm birth was also significant when using originally reported categories of duration of folate supplementation (<1, 1–2, 3–4, 5–6, 7–11, and >12 mo). A gradient is evident with less than 1 year of supplementation in the trend of survivor functions (test for trend of survivor functions *p* = 0.042) and proportion of spontaneous preterm birth before 32 wk (test for trend *p*<0.0001) (Figure 2).

Adjustment for the range of characteristics (maternal age, body mass index, race and ethnicity, educational level, marital status, smoking, parity and history of prior preterm birth) had only a minimal effect on the association between preconceptional folate supplementation for year or longer and spontaneous preterm birth at 20 to 28 wk and 28 to 32 wk. The adjustment for those characteristics eliminated significant association between shorter preconceptional folate supplementation for less than a year and spontaneous preterm birth (Table 2).

**Figure 3:**
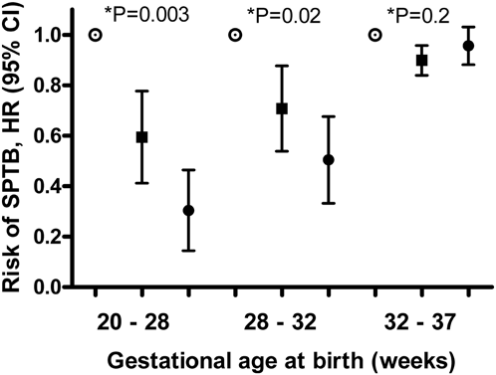
Relative risk of spontaneous preterm birth in relation to duration of preconceptional folate supplementation. HRs and 95% CIs for spontaneous preterm birth in women with preconceptional folate supplementation for a year or longer (•) or less than a year (▪), compared to women without preconceptional folate supplementation – reference value (○), from Cox proportional hazard regression. *Wald test for equality of hazard ratios, testing the joint hypothesis that coefficients are equal to 0 and that they are equal to each other [66]. SPTB, spontaneous preterm birth.

Figure 3 depicts the gradient in association between folate supplementation and risk of spontaneous preterm birth. There was a lower risk of spontaneous preterm birth for women with longer duration of preconceptional folate supplementation for preterm births between 20 and 28 wk and 28 and 32 wk. Duration of preconceptional folate supplementation was not associated with the risk of spontaneous preterm birth between 32 and 37 wk. The same gradient in the effect of duration of preconceptional folate supplementation on the risk of spontaneous preterm birth was observed when the three groups of the duration of preconceptional folate supplementation were coded as three levels of a single variable. The risk of spontaneous preterm birth at 20 to 28 and 28 to 32 wk was a function of the duration of preconceptional folate supplementation (adjusted HR 0.61, 95% confidence interval [CI] 0.41–0.93; *p* = 0.021 and 0.72, 0.54–0.96, *p* = 0.027, respectively). There was no significant association with the risk of spontaneous preterm birth at 32 to 37 wk (adjusted HR 0.99, 95% CI 0.92–1.07, *p* = 0.8).

**Figure 4:**
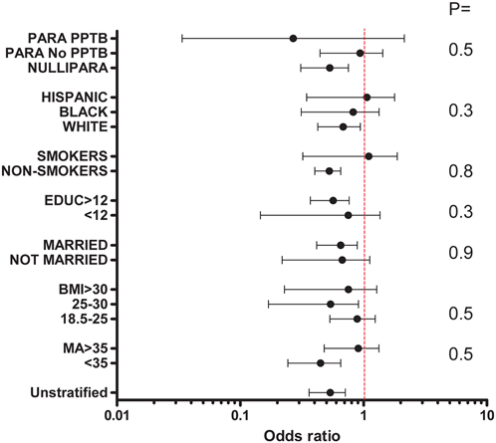
Stratified analysis of association between preconceptional folate supplementation ≥1 y and the risk of spontaneous preterm birth before 32 wk. ORs and 95% CIs of spontaneous preterm birth before 32 wk for preconceptional folate supplementation for ≥1 y, stratified for a given characteristic and adjusted for all other maternal characteristics. *p*-Values are estimated for interaction terms between duration of folate supplementation and given characteristic. The OR for BMI <18.5 could not be estimated, as there were no cases of spontaneous preterm birth before 32 wk among women using folate supplementation for ≥1 y and with BMI <18.5. MA, maternal age; Educ, completed years of education; PARA No PPTB, parous woman without a history of prior preterm birth; PARA PPTB, parous woman with a history of prior preterm birth.

There were no statistically significant interactions between duration of preconceptional folate supplementation and any of the maternal characteristics or risks factors for preterm birth in predicting the risk of spontaneous preterm birth (Figure 4). Additionally, in stratified analysis the point estimates of odds ratios (ORs) did not show a pattern indicative of interaction across strata of maternal characteristics and risk factors for preterm birth (Figure 4).

Body mass index (BMI), being married, and more than 12 y of education were associated with lower risk, while Hispanic ethnicity, black or Asian race, nulliparity, and prior preterm birth were associated with higher risk of spontaneous preterm birth (Table 3). The effects of Hispanic ethnicity, black or Asian race, nulliparity, and prior preterm birth were stronger for early spontaneous preterm birth. Maternal age and smoking did not have significant effects after adjustment for remaining characteristics (Table 3).

**Table 3:** Risk of spontaneous preterm birth in relation to maternal characteristics (*n* = 34,480).

Characteristic	Preterm Birth Gestational Age	*n* a	Preterm Birth		
			Adjusted HR	95% CI	*p*-Value
**Age**		(1,322/337)	1.01	0.89–1.14	0.9
**BMI**		(72/958/371/258)	0.93	0.87–0.99	0.02
**Race/Ethnicity**					
White			Reference		
Hispanic	20–28 wk	(21/41)	1.80	1.20–2.71	0.004
	28–32 wk	(37/62)	1.32	0.98–1.77	0.07
	32–37 wk	(309/1,189)	0.95	0.87–1.05	0.3
Black	20–28 wk	(14/48)	3.00	1.94–4.65	<0.0001
	28–32 wk	(9/90)	1.26	0.76–2.08	0.3
	32–37 wk	(96/1,402)	1.10	0.96–1.26	0.2
Asian	20–28 wk	(5/57)	2.08	1.19–3.63	0.01
	28–32 wk	(3/96)	0.80	0.38–1.71	0.6
	32–37 wk	(60/1,438)	1.00	0.87–1.15	0.9
**Married**		(409/1,250)	0.93	0.81–1.06	0.3
**Education**		(502/1,157)	0.87	0.77–0.98	0.034
**Smoking**		(1,561/98)	1.11	0.89–1.37	0.4
**Obstetrical history**					
Nulliparous	20–28 wk	(33/29)	2.37	1.31–3.31	0.005
	28–32 wk	(55/44)	2.38	1.55–3.74	<0.0001
	32–37 wk	(694/804)	1.46	1.30–1.64	<0.0001
Prior preterm birth	20–28 wk	(13/49)	5.46	2.62–11.36	<0.0001
	28–32 wk	(15/84)	4.10	2.22–7.58	<0.0001
	32–37 wk	(288/1,210)	4.38	3.79–5.06	<0.0001

HRs adjusted for maternal age, body mass index, race and ethnicity, educational level, marital status, smoking, parity, and history of prior preterm birth and preconceptional folate supplementation. Categorical variables: age, <35 (reference) versus ≥35 y; BMI <18.5 (reference), 18.5–25, 25–30, >30; married, not married (reference) versus married; education, ≤12 (reference) versus >12 y; smoking, nonsmoking (reference) versus smoking; nulliparous, nulliparous versus parous (reference); prior preterm birth, no prior preterm birth (reference) versus prior preterm birth. Proportional hazard assumption was met for each characteristic (proportional hazard test, *p*>0.05 for all).

a *n* = Number of spontaneous preterm births in each category in order as above or for dichotomous categories in order yes no.

There was no evidence of violation of the proportional hazard assumption for each variable (*p*>0.05 for all) or globally for the whole model (*p* = 0.7). The goodness of fit test showed no evidence of poor fit (*p* = 0.5)

The proportion of the PPROM among folate supplementation groups in women with spontaneous preterm birth before 32 wk was not significantly different (20.0%, 41.3%, and 36.4%, *p* = 0.3; for folate supplementation for ≥1 y, <1 y, and no folate supplementation, respectively).

There was no significant association between duration of preconceptional folate supplementation and the risk of preeclampsia or placental abruption. The risk of delivery of a small for gestational age infant was not significant after adjustment for maternal characteristics (Table 4).

**Table 4:** Risk of SGA infant, placental abruption, and preconceptional folate supplementation.

Risk	No Folate	Folate <1 y	Folate ≥1 y	Folate <1 y OR (95% CI)				Folate ≥1 y OR (95% CI)			
	No. (%) (*n* = 15,259)	No. (%) (*n* = 12,444)	No. (%) (*n* = 6,777)	Unadjusted	*p*-Value	Adjusted	*p*-Value	Unadjusted	*p*-Value	Adjusted	*p*-Value
**SGA infant**	1,556 (10.2)	1,081 (8.7)	582 (8.6)	0.83 (0.77–0.91)	<0.001	0.93 (0.85–1.02)	0.1	0.82 (0.75–0.91)	<0.001	0.96 (0.86–1.08)	0.5
**Placental abruption**	107 (0.70)	91 (0.73)	50 (0.74)	1.04 (0.79–1.47)	0.3	0.86 (0.64–1.17)	0.3	1.05 (0.75–1.48)	0.3	0.80 (0.55–1.14)	0.2
**Preeclampsia**	381 (2.2)	295 (2.2)	157 (2.1)	0.96 (0.83–1.12)	0.6	1.03 (0.86–1.23)	0.9	0.94 (0.78–1.13)	0.5	1.05 (0.85–1.31)	0.7

No Folate, no preconceptional folate supplementation; Folate <1 yr, preconceptional folate supplementation for <1 year; Folate ≥1 yr, preconceptional folate supplementation for ≥1 year.

Adjusted for maternal age, body mass index, race and ethnicity, educational level, marital status, smoking, parity and history of prior preterm birth.

SGA, small for gestational age.

Among patients excluded from analysis who had data on preconceptional folate supplementation, folate was used by 521 (14.7%) for 1 y or longer and by 1,018 (28.6%) for less than 1 y, and was not used by 1,827 (51.4%).

## Discussion

### Interpretation of the Key Findings

We have shown that preconceptional folate supplementation for a year or longer is associated with a decreased risk of early spontaneous preterm birth. The association is characterized by strength, temporal relationship, biological gradient, nonsignificant effect of confounders, consistency with other studies, and biological plausibility.

Preconceptional folate supplementation for a year or longer was associated with over a 70% reduction in the risk of spontaneous preterm birth before 28 wk and 50% reduction of the risk of spontaneous preterm birth between 28 and 32 wk.

The strength of the association was significantly weaker if folate supplementation was shorter than a year. There was also a significant trend of decreased risk of spontaneous preterm birth across the original seven categories of duration of folate supplementation. This indicates existence of a biological gradient in the association between preconceptional folate supplementation and the risk of spontaneous preterm birth.

The relationship between preconceptional folate supplementation and risk of spontaneous preterm birth could potentially be confounded by risk factors for spontaneous preterm birth and indications for preterm delivery. However, adjustment for the risk factors for spontaneous preterm birth had a minimal effect on the association. The change in the coefficients observed with adjustment was 32% for spontaneous preterm birth between 20 and 28 wk and 26% for spontaneous preterm birth between 28 and 32 wk. The resulting reduction in the risk of spontaneous preterm birth, due to adjustment, of 78% versus 69% or of 55% versus 47%, respectively, is clinically of no material importance.

Pregnancies with indications for preterm delivery were censored, contributing to the denominator, the number of pregnancies at risk, but not included in the nominator, the number of spontaneous preterm births at each gestational age time point. Analysis without censoring showed a significant association between preconceptional folate supplementation and lower risk of all early preterm births. However, there was no significant association between duration of folate supplementation and the risk of nonspontaneous preterm birth.

There were no significant interactions between folate supplementation and maternal charactersitics and risk factors for preterm birth, indicating that this association is observed regardless of risk factors for preterm birth. Although the confidence intervals of some of the strata were large and crossed unity due to a small size of the strata, the interaction terms of these covariates and duration of folate supplementation, which do not depend on the size of the strata, are not significant.

The findings of this study are consistent with prior observational studies showing that women delivering prematurely have lower folate concentrations in plasma or red blood cells than women delivering at term [8],[24]–[26]. The interventional trials of folate supplementation during pregnancy resulted in longer duration of pregnancy in some [11],[27] but not in all studies [28]–[30]. However, those trials evaluated folate supplementation during pregnancy, most in the third trimester rather than preconceptionally, and were of insufficient size to assess early preterm birth. Trials of folate supplementation for prevention of neural tube defects did not evaluate the risk of preterm birth and had similar limitations [31]–[35]. However, since 1998 there has been a compulsory fortification of flour and grains with folate in the US. Thus all women enrolled in the study were exposed to mandatory folate fortification of grains. The prevalence of singleton preterm deliveries decreased after the compulsory fortification was instituted [36]. However, the decline in the incidence of preterm birth and neural tube defects was substantially less after fortification was instituted than was expected from the clinical trials [37],[38]. This was attributed to insufficient amount of folate supplementation provided by folate grain fortification.

A substantial body of evidence suggests that the association between folate supplementation and spontaneous preterm birth is biologically plausible. Spontaneous preterm birth, especially at early gestation, is strongly associated with intrauterine infection [39]. Impaired function of neutrophils and lymphocytes, and increased prevalence of bacteriuria in pregnancy (a risk factor for preterm birth) are associated with low plasma folate concentrations [40]–[42]. Moreover, in a randomized trial, folate supplementation was shown to decrease markers of inflammation during pregnancy [43]. Recent findings have also shown an association between polymorphism or deletion in folate-metabolizing genes and the risk of preterm birth [44],[45]. Biological plausibility of intrauterine infection–mediated effect of folate supplementation is also supported by lack of association with late spontaneous preterm birth. Late spontaneous preterm birth is much less strongly associated with intrauterine infection than is spontaneous preterm birth before 32 wk of pregnancy [46].

### Limitations of the Analyses

Early spontaneous preterm birth is a devastating but rare outcome. Thus the findings of this study are based on small number of cases, and additional cases may change substantially those estimates in either direction. This is reflected in their wide confidence intervals. However, the estimates remain statistically significant also using exact logistic and Poisson regressions methods, which are accurate with rare outcomes (Table S1).

A limitation of observational study design is a possibility that relevant confounders have not been accounted for. Thus it is conceivable that folate supplementation could be a marker of healthy behavior. Such an alternative hypothesis is unlikely because the association between folate supplementation and the risk of spontaneous preterm birth is specific to spontaneous preterm birth, is not affected by other markers of healthy behavior, and depends on the length of supplementation in a similar fashion to the effect of folate supplementation on other disorders observed in randomized trials.

Preconceptional folate supplementation was associated specifically with the risk of early spontaneous preterm birth and not with the risk of other complications of pregnancy, preeclampsia, small for gestational age infant, placental abruption, or nonspontaneous preterm birth. Markers of healthy behavior, maternal characteristics associated with healthy behavior, maternal age, BMI, education, marital status, and smoking minimally affected the association between folate supplementation and the risk of spontaneous preterm birth. If preconceptional folate supplementation were a mere marker of healthy behavior, adjustment for other markers of healthy behavior would either eliminate or significantly attenuate the observed association with the risk of spontaneous preterm birth. In fact, in our study, adjustment for markers of healthy behavior did not have a substantial effect.

Thus it is unlikely that unaccounted confounding could eliminate observed associations because of the specificity of this association with only early spontaneous preterm birth and because two unaccounted confounders—markers of socioeconomic status or healthy behavior—are strongly correlated with measured confounders [1]. Moreover, some of the confounders included in the analysis may be on the mechanistic pathway of the folate effect.

Short interpregnancy interval and unintended pregnancy are unlikely to confound the findings, because the association between preconceptional folate supplementation and risk of spontaneous preterm birth was significant also in nulliparous women and largely independent of maternal age, race/ethnicity, marital status, and education, known risk factors for unintended pregnancy [47]. Conversely, subfertility is associated with multiple complications of pregnancy such as preeclampsia and delivery of small for gestational age infant, which were not associated with folate supplementation [48].

None of the above-mentioned observations is sufficient by itself to exclude the existence of unaccounted confounders, such as healthy behavior of women who use folate preconceptionally. However, collectively they strongly support a nonspurious association of preconceptional folate supplementation and the risk of spontaneous preterm birth. Definitive support for this hypothesis can be provided only by a randomized trial.

The importance of duration rather than dosage of folate supplementation has been observed previously. Meta-analyses of folate for prevention of neural tube defects or stroke did not show a significant association between dosage of folate supplementation and the strength of its effect [49],[50]. Meta-analysis of randomized controlled trials of folate supplementation in stroke prevention has shown that an inverse relationship exists between the duration of folate supplementation and the risk of stroke, and that duration rather than dosage of folate supplementation is critical [51].

Finally, women who used folate supplementation preconceptionally had a lower risk of spontaneous preterm birth than women who did not use it, regardless of the unknown dosage and frequency of their use.

Use of folate in a combination with other micronutrients could potentially confound the findings. However, a systematic review and individual clinical trials have shown that supplementation with multiple micronutrients in pregnancy did not offer additional benefit over folate and iron supplementation alone [52]–[54]. In fact, supplementation with some micronutrients, such as vitamins C and E, was associated with increased risk of low birth weight [55].

Supplementation with zinc has been associated with lower risk of preterm birth [56]. However, this effect was observed primarily in the studies of low-income women. Conversely, in this study, folate supplementation was associated with a lower risk of spontaneous preterm birth regardless of the markers of socioeconomic status. Low zinc levels were also associated with placental abruption, a relationship that was not evident in this study of folate supplementation [57]. Moreover, data suggest that adding zinc may negate the beneficial effect of folate and iron on low birth weight [58],[59].

Postconceptional folate supplementation also could not confound our findings. In the US 93% of women use postconceptional folate supplementation and in a population with early onset of prenatal care (as in this study) the rate is likely much higher [60]. Moreover, a systematic review of folate supplementation during pregnancy has shown no significant effect on the risk of preterm birth, duration of pregnancy, or birth weight [61].

Another limitation of this study is that preconceptional folate supplementation was self-reported at enrollment in the first trimester of pregnancy. However, self-reported folate intake from supplements has been shown to correlate with serum folate concentrations during pregnancy and was a reliable measure of folate supplementation [62]. Additionally, duration of preconceptional folate supplementation was reported prospectively, and its rate is similar to that in the general population [63]. Although a significant reduction in the risk of spontaneous preterm birth was observed across the original categories of duration of folate supplementation, we analyzed larger categories, that are consistent with other studies, and allowed analysis of confounders and interactions [64].

### Implications and Generalizability

The validity and generalizability of these findings are supported by a large number and proportion of patients included in the analysis. Less than 10% of patients were excluded from the multivariable analysis. Distribution of the length of folate supplementation in the excluded pregnancies was similar to that observed in the study population. Moreover, the study population is a good representation of a general US population [65]. The incidence of early preterm birth the main outcome of the study is 1.5% in the general population and 1.4% in the study population [1]. There was no significant association between preconceptional folate supplementation and duration of pregnancy, because early spontaneous preterm birth contributes only a very small fraction to the duration of pregnancy. Univariate association with the birth weight is likley a result of confounding by risk factors for fetal growth restriction, because the association was eliminated after adjustment for maternal characteristics.

We have shown that preconceptional folate supplementation is associated with a 50%–70% reduction in the incidence of early spontaneous preterm birth. These findings, despite their limitations, provide a basis for further inquiry into preconceptional folate supplementation for prevention of spontaneous preterm birth in clinical trials.

## Supporting Information

Table S1 **Risk of spontaneous preterm birth and preconceptional folate supplementation: Results of maximum likelihood and exact models.** Definitions: No folate, no preconceptional folate supplementation; folate <1 y, preconceptional folate supplementation for <1 year; folate ≥1 yr, preconceptional folate supplementation for ≥1 y. HR 95% CI is from Cox regression with Breslow assumption maximum likelihood model; OR 95% CI, from exact logistic regression; IRR (incidence rate ratio) 95% CI, from exact Poisson regression.
(0.04 MB DOC)

Table S2 **Participating centers.**
(0.02 MB DOC)

## Abbreviations

BMI: body mass index
CI: confidence interval
HR: hazard ratio, IQR, interquartile range
OR: odds ratio
PPROM: preterm premature rupture of the membranes
