# Supplementary material for: Preconceptional Folate Supplementation and the Risk of Spontaneous Preterm Birth: A Cohort Study
Source: PLoS Med. 2009 May 12;6(5):e1000061. doi: 10.1371/journal.pmed.1000061 (PMC2671168; doi:10.1371/journal.pmed.1000061)
Supplement: Table S1 — Risk of spontaneous preterm birth and preconceptional folate supplementation: Results of maximum likelihood and exact models. Definitions: No folate, no preconceptional folate supplementation; folate <1 y, preconceptional folate supplementation for <1 year; folate ≥1 yr, preconceptional folate supplementation for ≥1 y. HR 95% CI is from Cox regression with Breslow assumption maximum likelihood model; OR 95% CI, from exact logistic regression; IRR (incidence rate ratio) 95% CI, from exact Poisson regression. (0.04 MB DOC) [file pmed.1000061.s001.doc]

**Table S1. Risk of spontaneous preterm birth and preconceptional folate supplementation: results of maximum likelihood and exact models.**

| **Preterm birth** | **Folate <1 yr** | | **Folate >1 yr** | |
| --- | --- | --- | --- | --- |
|  | **HR (95% CI)** | **P** | **HR (95% CI)** | **P** |
| **20-28 wks** | 0.54  (0.31-0.93) | 0.028 | 0.22  (0.08-0.61) | 0.004 |
| **28-32 wks** | 0.67  (0.44-1.02) | 0.061 | 0.45  (0.24-0.83) | 0.010 |
|  | **OR (95% CI)** | **P** | **OR (95% CI)** | **P** |
| **20-28 wks** | 0.51  (0.27-0.91) | 0.022 | 0.17  (0.03-0.52) | 0.0003 |
| **28-32 wks** | 0.63  (0.39-0.99) | 0.045 | 0.46  (0.23-0.87) | 0.013 |
|  | **IRR (95% CI)** | **P** | **IRR (95% CI)** | **P** |
| **20-28 wks** | 0.51  (0.27-0.91) | 0.021 | 0.17  (0.03-0.51) | 0.0003 |
| **28-32 wks** | 0.63  (0.39-0.99) | 0.044 | 0.46  (0.23-0.87) | 0.013 |

Abbreviations: No Folate, no preconceptional folate supplementation; Folate <1 yr, preconceptional folate supplementation for < 1 year; Folate >1 yr, preconceptional folate supplementation for > 1 year;

HR, Hazard ratio; 95 % CI, 95 % confidence interval from Cox regression with Breslow assumption maximum likelihood model;

OR, Odds ratio; 95 % CI, 95 % confidence interval from exact logistic regression;

IRR, Incidence rate ratio; 95 % CI, 95 % confidence interval from exact Poisson regression;
