# Supplementary material for: Preconceptional Folate Supplementation and the Risk of Spontaneous Preterm Birth: A Cohort Study
Source: PLoS Med. 2009 May 12;6(5):e1000061. doi: 10.1371/journal.pmed.1000061 (PMC2671168; doi:10.1371/journal.pmed.1000061)
Supplement: Table S2 — Participating centers. (0.02 MB DOC) [file pmed.1000061.s002.doc]

**Table T 2. Participating centers.**

1. University of Texas Medical Branch, Galveston, Texas
2. University of Utah, Salt Lake City, Utah (4 centers)
3. Swedish Medical Center, Seattle, Washington
4. William Beaumont Hospital, Fetal Imaging Department, Royal Oak, Michigan
5. Mount Sinai Medical Center, New York, New York
6. Montefiore Medical Center/Albert Einstein College of Medicine, Bronx, New York
7. University of Colorado Health Sciences Center, Denver, Colorado ;
8. Tufts-New England Medical Center, Boston, Massachusetts ;
9. NYU Medical Center, School of Medicine New York, New York
10. Women and Infants' Hospital / Brown University, Providence, Rhode Island
11. University of North Carolina at Chapel Hill, Chapel Hill, North Carolina
12. Columbia University, New York, New York
